# Health Effects of Ergonomics and Personal Protective Equipment on Chemotherapy Professionals

**DOI:** 10.3390/curroncol32100563

**Published:** 2025-10-08

**Authors:** Ana Reis, Vítor Silva, João José Joaquim, Luís Valadares, Cristiano Matos, Carolina Valeiro, Ramona Mateos-Campos, Fernando Moreira

**Affiliations:** 1 Facultad de Farmacia, Universidad de Salamanca, Campus Miguel de Unamuno, 37007 Salamanca, Spain; ritareis3@gmail.com; 2 Unidade Local de Saúde de Coimbra, Praceta Prof. Mota Pinto, 3000-075 Coimbra, Portugal; 16314@ulscoimbra.min-saude.pt; 3 Associação Portuguesa de Licenciados em Farmácia (APLF), 3140-348 Coimbra, Portugal; jjj@estesc.ipc.pt; 4 Farmácia, Escola Superior de Tecnologia da Saúde de Coimbra, Instituto Politécnico de Coimbra, R. 5 de Outubro, 3045-043 Coimbra, Portugal; 5 Unidade Local de Saúde (ULS) São José E.P.E, 1150-199 Lisbon, Portugal; luis.valadares@ulssjose.min-saude.pt; 6 European Association of Pharmacy Technicians, B-1080 Brussels, Belgium; carbenval@alum.us.es; 7 Área de Medicina Preventiva y Salud Pública, Departamento Ciencias Biomédicas y del Diagnóstico, Faculdade de Medicina, Universidad de Salamanca, 37007 Salamanca, Spain; rmateos@usal.es; 8 LAQV/REQUIMTE, Escola Superior de Saúde, Instituto Politécnico do Porto, Rua Dr. António Bernardino de Almeida, 4200-072 Porto, Portugal

**Keywords:** cytotoxic drugs, contamination, personal protective equipment, ergonomic practices

## Abstract

**Simple Summary:**

Chemotherapy drugs are vital for treating cancer, but the professionals who prepare and administer them may be exposed to small amounts that can harm their health over time. To stay protected, they use personal protective equipment like gloves, gowns, and masks, and must also work under ergonomic conditions that prevent strain from repetitive tasks and awkward postures. This review examined recent studies to see how these protective measures are applied and what risks remain. We found that use of protective equipment is often inconsistent and ergonomic challenges are common, especially for nurses and pharmacy technicians. These findings show the importance of better training, safer workplace design, and stronger institutional support. Improving protective practices and ergonomics can help protect healthcare workers, enhance their wellbeing, and ensure safer patient care.

**Abstract:**

(1) Background: With the increasing incidence of cancer, the need for handling cytotoxic drugs has also grown. However, manipulating these drugs exposes healthcare professionals to significant risks, including occupational exposure to hazardous chemicals. Therefore, it is important to adopt protective measures, including personal protective equipment (PPE) and correct ergonomic practices, to ensure safe drug preparation and minimize health risks for the operators. However, while chemical exposure and PPE have been extensively addressed in the literature, the combined impact of ergonomic practices and protective measures remains insufficiently emphasized, representing a critical gap this review aims to address. Accordingly, the objective of this literature review was to analyze the ergonomic and individual protection practices during the handling of cytostatic drugs and all the implications that bad ergonomic practices and/or poor individual protection have on the operator’s health; (2) Methods: In order to perform this integrative review, a structured literature search was conducted using online databases (Web of Science^®^, Google Scholar^®^, and PubMed^®^) from January 2005 to June 2025. (3) Results: A total of 19 articles were analyzed, with 17 focusing on PPE and 17 on ergonomics. The findings emphasize that PPE, such as gloves, masks, gowns, sleeves and safety glasses, plays a critical role in the safe handling of cytotoxic drugs, particularly when combined with other safety measures. Additionally, maintaining correct ergonomic posture is important in preventing musculoskeletal disorders; (4) Conclusions: This review emphasizes the significance of integrating appropriate PPE use with sound ergonomic procedures. Although PPE is still the secondary line of defense against occupational exposure, ergonomic issues must also be addressed to avoid chronic musculoskeletal problems. Continuous training, rigorous attention to safety procedures, and ergonomic enhancements should be prioritized by healthcare facilities as a key element of occupational safety programs to reduce the short-term and long-term health hazards for personnel handling dangerous drugs.

## 1. Introduction

Cytotoxic drugs, also known as antineoplastics, are a class of medications that contain chemicals toxic to cells, primarily used in the treatment of cancer. Their use has been increasingly common, not only for the therapy of malignant diseases but also for prophylactic purposes. Furthermore, their use has expanded to an increasing spectrum of benign pathologies, such as autoimmune diseases and chronic inflammatory conditions, particularly in gastroenterology and rheumatology. These agents work by inhibiting cell replication and growth, which is essential in targeting malignant cells [1]. However, their action is not exclusively targeted and, as a result, they can also affect normal, healthy cells, leading to various side effects in patients and potentially exposing others to these toxic effects [2,3], including healthcare professionals who handle these substances [4]. In oncology, the therapeutic use of these drugs requires careful selection of the combination of agents, dosage, and treatment regimen, since the concept of selectivity cannot be applied—all chemotherapy drugs inevitably produce adverse effects and impact normal cells [5]. These harmful effects on healthy tissue may include hair loss, skin rashes, infertility, spontaneous abortions, and congenital malformations [6,7,8,9]. Despite these side effects and associated risks, cytotoxic drugs remain highly beneficial for cancer patients, as they help eliminate malignant cells, prolong survival, and improve quality of life. Thus, chemotherapy remains the first-line treatment for most types of cancer [10].

Concerns about occupational exposure to cytotoxic drugs were first raised by Falck et al., in 1979 [11]. In the context of occupational health, these drugs are classified as hazardous drugs—agents whose inherent toxicity poses risks to healthcare professionals [12]. Nevertheless, occupational exposure is often not adequately controlled or prevented. The likelihood of occupational exposure increases when control measures are inadequate. The main routes of exposure include skin contact, skin absorption, inhalation of aerosols and drug particles, ingestion, and needlestick injuries. These risks can arise during drug preparation, administration, handling of patient waste, transport, waste disposal, or cleaning of spills [13]. Inadequate or insufficient control measures can lead to health issues such as abdominal pain, hair loss, nasal sores, vomiting, contact dermatitis, localized allergic reactions and liver damage [14,15,16,17]. Other adverse effects include fetal loss in pregnant women, and congenital malformations in their children [18,19]. Furthermore, exposure to cytotoxic drugs can alter normal blood cell counts and induce mutagenic activity, leading to abnormal cell formation [20,21,22,23]. Corroborating these findings, the International Agency for Research on Cancer (IARC) has classified many of these drugs as carcinogenic, mutagenic, and/or toxic for reproduction [24].

Those at risk include medical, pharmacy technicians, pharmacists, nursing staff, laboratory workers, cleaning personnel, patients’ family members and caregivers, as well as the patients themselves [25,26,27]. Healthcare professionals involved in the preparation and administration of these drugs (pharmacists, pharmacy technicians and nurses) are at increased risk of experiencing adverse health effects. Previous studies have demonstrated widespread contamination of the environment and work surfaces in healthcare settings [4,28,29,30]. Therefore, it is essential to implement and maintain effective control measures to protect these professionals from exposure.

Biological Safety Cabinets (BSC) are a key engineering control that provide containment during the preparation of cytotoxic drugs [31]. The BSCs help minimize the risk of aerosolization and environmental contamination, and thus reduce exposure to hazardous drug vapors and particles while also providing an aseptic environment [32]. When used in conjunction with appropriate PPE, BSCs form part of a comprehensive safety strategy for mitigating occupational risks.

Regular training regarding the risks and safe handling of cytotoxic drugs is also an important consideration. Healthcare workers, especially pharmacy technicians, pharmacists, and nurses, who are often responsible for the reconstitution, preparation, dilution, and mixing of cytotoxic drugs, must receive proper training and be adequately equipped to work under aseptic conditions [31,32]. PPE plays a pivotal role in reducing the probability of exposure to cytotoxic agents, particularly through dermal contact [33]. Appropriate PPE includes chemical-resistant gloves, impervious gowns, P2 or P3 masks, safety goggles or face shields, caps, and shoe covers—each designed to prevent skin and mucosal exposure to hazardous substances. Since the exposure to cytotoxic drugs can occur even in very small quantities; PPE represents one of the most critical lines of defense in protecting professionals from potentially serious health effects [29,34,35].

Control measures focus on reducing the quantities of drugs used, limiting the number of exposed employees, and minimizing the duration of exposure [6]. Safe handling practices, proper storage, and appropriate disposal of cytotoxic drugs along with contaminated waste are necessary to prevent accidental exposure [36]. Maintaining good hygiene practices is also crucial—for example, prohibiting eating, drinking, and smoking in areas where these drugs are handled, as well as ensuring access to adequate washing facilities [31,37,38].

Clinical practice and oncology hospitals, pharmacies, and caregiver organizations have established strict guidelines to protect healthcare professionals from occupational exposure to cytotoxic drugs and to ensure that contaminated materials are correctly disposed of [4,39]. These recommendations are particularly important given the risks of harmful exposure during the preparation, administration, and waste management of such agents. In addition, while pharmaceutical companies and regulatory agencies receive continuous updates regarding the classification and management of cytotoxic drugs, scientific data and risk assessments have increasingly served as a foundation for establishing exposure limits, safety standards, and handling protocols [4,40,41].

In the context of occupational exposure to cytotoxic drugs, several international and European frameworks provide overarching guidance. Globally, agencies such as the National Institute for Occupational Safety and Health (NIOSH) and the Occupational Safety and Health Administration (OSHA) in the United States have issued foundational recommendations on the safe handling of hazardous drugs, focusing on engineering controls, safe work practices, and appropriate use of PPE [12,42]. Complementing these, the International Society of Oncology Pharmacy Practitioners (ISOPP) has developed practice standards specifically tailored to oncology pharmacy settings [31]. In Europe, regulatory emphasis is placed on preventing exposure through manufacturing and workplace controls. The European Medicines Agency (EMA) provides Good Manufacturing Practice (GMP) guidelines addressing containment and cross-contamination [43], while the EU-OSHA Carcinogens and Mutagens Directive (2004/37/EC, amended by 2022/431) requires employers to minimize workers’ exposure to hazardous medicinal products, including cytotoxics [44]. Individual Member States, such as the UK (HSE/COSHH) [45] and Germany (BAuA) [46], further operationalize these principles through national regulations. Taken together, these international and European frameworks share a common goal: minimizing occupational risks associated with cytotoxic drugs by combining risk assessment, containment systems, and protective measures, while differing mainly in scope—practice standards in the US, EU-wide legislation in Europe, and more detailed national adaptations at the country level.

Ergonomics, broadly defined as the alignment of job demands with workers’ capabilities and workplace design, has traditionally focused on preventing work-related musculoskeletal disorders, which remain the most common occupational health issue across sectors [47]. By optimizing efficiency, comfort, and safety, ergonomics not only reduces physical strain and injury but also improves productivity, satisfaction, and workforce retention. Risk factors for work-related musculoskeletal disorders include repetitive or forceful movements, awkward or sustained postures, heavy lifting, and inadequate equipment, with frequent consequences such as back, neck, and upper extremity disorders [48,49]. Evidence highlights that psychosocial factors—such as job stress, work organization, and lack of social support—also interact with physical risks, exacerbating injury rates and contributing to burnout, absenteeism, and psychological distress [50,51]. This is particularly relevant in healthcare, where long working hours, high physical demands, and poor ergonomic arrangements heighten both physical and psychosocial risks. Despite these concerns, ergonomics is often underrepresented in the occupational safety literature compared to chemical hazards and PPE. Addressing this gap is essential, as inadequate ergonomic conditions not only compromise worker health but also undermine overall quality of care and organizational performance.

Accordingly, in addition to the need for strict measures to minimize exposure, ergonomic considerations are also important in ensuring the safety of pharmacy technicians [52]. In particular, poor ergonomics during the preparation of cytotoxic drugs can result in musculoskeletal disorders, including pain and stiffness in the arms, shoulders, neck, and back [26,53,54].

The confined space of a BSC, combined with repetitive and conditioned motions the exertion required for certain tasks, can lead to long-term health issues (such as leading to osteoarticular injuries, muscle pain) [55]. Therefore, ergonomic measures must be implemented to prevent such injuries and improve task efficiency, ensuring both safety and comfort for healthcare professionals [55,56].

Although ISOPP recommendations underscore the relevance of maintaining appropriate temperature, humidity, and ventilation to safeguard the comfort of healthcare professionals handling cytotoxic drugs, the vast majority of international guidelines remain silent on ergonomics [31].

The handling of cytotoxic drugs presents not only chemical hazards but also significant physical and ergonomic challenges for healthcare professionals, particularly pharmacy technicians and nurses. While much attention has been given to chemical exposure risks, the importance of proper use of PPE and ergonomic practices is sometimes underestimated. Incorrect or inconsistent use of PPE can result in direct exposure to hazardous substances, while poor ergonomic conditions—such as prolonged awkward postures, repetitive motions, and working within confined spaces like BSC—can lead to musculoskeletal disorders and chronic health problems. Understanding and improving these factors is critical to ensuring the overall safety and wellbeing of personnel involved in cytotoxic drug preparation and administration, reducing both acute toxic risks and long-term occupational injuries. Therefore, assessing PPE adherence and ergonomic factors in this context is essential to inform effective interventions and promote safer work environments. Previous reviews have mainly focused on chemical hazards and PPE use, overlooking ergonomic risks in the handling of cytotoxic drugs [57,58,59,60]. For example, while some emphasized barriers to PPE compliance or the importance of contamination monitoring, none systematically addressed ergonomics as part of occupational safety. By combining PPE and ergonomics, this review provides a more comprehensive perspective and responds to an important gap in the existing literature.

Therefore, this review aims to discuss the importance of PPE and proper ergonomic practices in safeguarding healthcare workers involved in tasks related to the handling of cytotoxic agents, as well as the potential health risks they may face if appropriate precautions are not followed.

## 2. Materials and Methods

This study was designed as an integrative review, as it combines evidence from original research and review articles, synthesizing findings across diverse study designs to provide a comprehensive understanding of the use of PPE and ergonomic challenges in the handling of cytotoxic drugs. The aim was to capture and integrate the most relevant evidence to contextualize current practices and knowledge gaps. The primary databases used were PubMed^®^, Google Scholar^®^, and Web of Science^®^. Combinations of the following keywords were used to select relevant references: “Cytotoxic Drugs,” “Handling”, “Musculoskeletal injuries”, “Personal Protective Equipment,” and “Ergonomics”. The complete search strings presented in Table 1 were applied.

The inclusion criteria comprised articles published from 1 January 2005 to 30 June 2025—written in English and focused on the handling of cytotoxic drugs, the importance of PPE, or ergonomic challenges. The last search through all databases was performed on 1 July 2025. Peer-reviewed original research studies were prioritized, but systematic and narrative reviews relevant to the topic were also included to provide a broader synthesis of existing evidence. Articles that lacked scientific relevance to the research question were excluded based on title and abstract analysis. Additionally, non–peer-reviewed publications (such as conference abstracts), editorials, guidelines and opinion pieces, were also excluded.

To minimize the risk of omitting relevant studies, the reference lists of selected articles were reviewed.

The results were synthesized using a thematic analysis approach. Studies were classified into two main thematic axes: (1) Personal Protective Equipment (PPE), including training, compliance, and use of specific protective devices; and (2) Ergonomics, encompassing musculoskeletal risks, workspace design, and preventive measures.

No formal critical appraisal of methodological quality was performed; however, studies were selected based on scientific relevance, peer-reviewed status, and methodological transparency to ensure reliable synthesis.

## 3. Results

**Figure 1:**
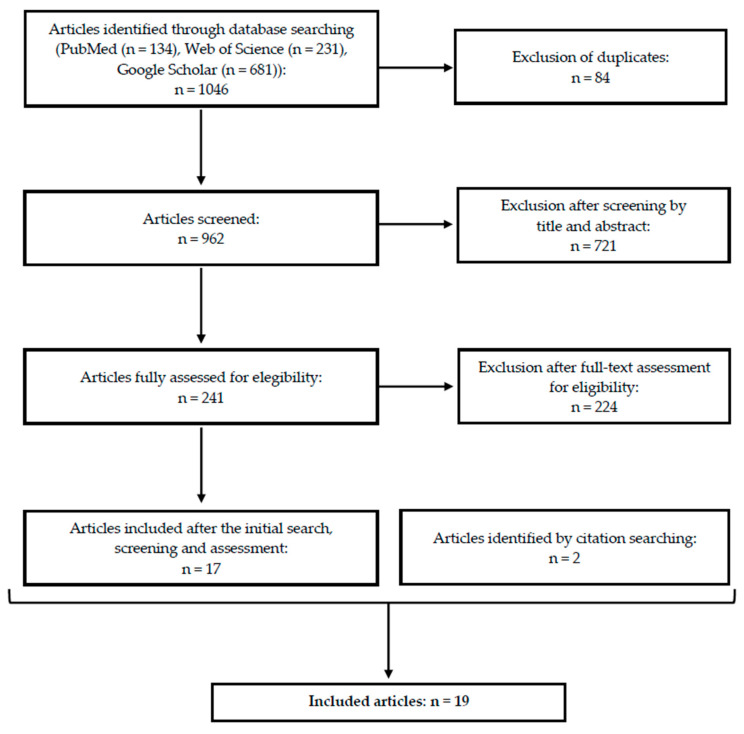
Simplified flow diagram of the literature search and selection process—number of records identified, screened, excluded, and finally included in the integrative review.

After the identification of 1046 articles during the primary search, a total of 19 articles were ultimately included (Figure 1).

Fifteen of the 19 included studies (79%) addressed both PPE use and ergonomics [6,29,33,57,61,62,63,64,65,66,67,68,69,70,71], two (10.5%) focused solely on PPE [72,73], and the remaining two (10.5%) exclusively on ergonomics [56,74]. Most of the studies described observational or experimental methodologies that are depicted on Table 2.

Different types of reviews were deemed relevant during the selection of published papers, including narrative, scoping and systematic reviews (Table 3).

**Figure 2:**
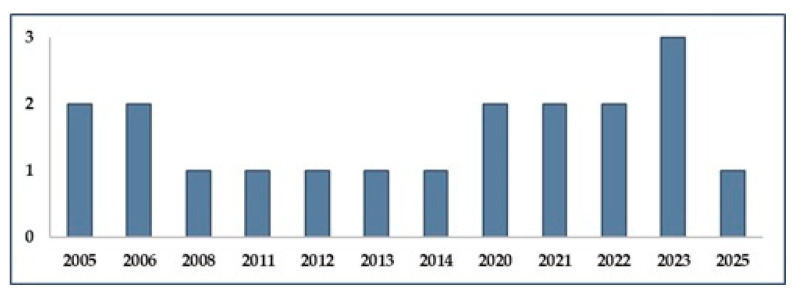
Year of publication of the 19 studies included in the review, showing the temporal distribution of research on PPE and ergonomics in the handling of cytotoxic drugs.

The temporal distribution of the included studies is presented in Figure 2, which display the publication years.

**Figure 3:**
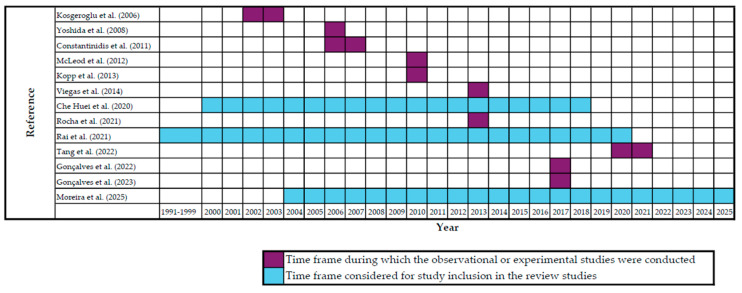
Time frames covered by the included studies, distinguishing observational/experimental studies from review studies [6,29,33,56,57,62,63,64,65,66,67,68,69].

Thirteen of the 19 included studies described their occurrence over time, which is further demonstrated in Figure 3, according to the research periods covered.

The main findings of the included studies regarding the use of personal protective equipment and ergonomic aspects are summarized in Table 4 and Table 5, respectively.

## 4. Discussion

### 4.1. Personal Protective Equipment (PPE)

PPE remains a cornerstone in the protection of healthcare workers against cytotoxic drug exposure, but significant lapses in correct selection, use, and replacement persist across studies [61,70]. Gloves are the most consistently used item, especially during drug preparation and administration [33,63]. However, issues remain regarding glove type, thickness, permeation resistance, and replacement frequency. Vinyl gloves, for instance, allow rapid permeation of several cytotoxic agents, whereas latex, nitrile, or neoprene gloves offer superior resistance, particularly when at least 0.2 mm thick. To ensure adequate protection, gloves should be changed every 15–30 min, or immediately if torn or contaminated, with double-gloving recommended during high-risk tasks such as drug preparation [2,31,61,63,70]. Despite these guidelines, adherence is inconsistent. Kopp et al. [63] found that while gloves were worn by 92.5% of staff during drug preparation, use dropped sharply during unpacking (2.5%) and surface cleaning (35%). Similarly, Yoshida et al. [33] reported that only 29.2% of facilities practiced double-gloving, and 10.1% reported not using PPE at all for certain tasks.

Gowns are another critical component, intended to be single-use, fluid-resistant, and made from non-woven, low-permeability materials with long sleeves and tight cuffs [32]. Nonetheless, Gonçalves et al. [69] observed instances of gowns being reused to reduce costs, particularly in resource-limited hospitals, directly contravening safety standards. Respiratory protection shows similar gaps: although FFP2 (or higher) respirators are recommended for aerosol-generating procedures, many facilities rely on surgical masks, which are ineffective in this context [63,66,68,69,73]. Moreover, staff are often unaware of the importance of proper fit when using respirators [68,69,72]. Eye and face protection is also underused, often due to discomfort. When worn, devices are sometimes inadequate—for example, simple plastic visors with poor lateral coverage—leaving workers insufficiently protected from splashes [12,31,33,63,68]. Foot protection, such as overshoes, is rarely implemented despite recommendations to reduce contamination risks [32,75].

Another widespread issue is the extended use or reuse of disposable PPE, reported in both high- and low-resource settings [6,69]. This practice compromises barrier integrity and increases the likelihood of cross-contamination between environments and patients. Training deficiencies compound these risks: Kopp et al. [63] reported that only 25% of institutions provided hands-on training in PPE use, and even fewer had procedures for safe disposal or spill response. Consequently, healthcare workers often rely on informal instruction or habits rather than standardized protocols [57].

Several studies also highlighted institutional and organizational variation influencing PPE use. For example, McDiarmid & Condon [72] and Yoshida et al. [33] reported wide differences in the implementation of safety practices across hospitals, particularly smaller ones. Fransman et al. [73] further noted that hospital layouts and glove availability limited comparability across settings, while Kopp et al. [63] described preparation practices outside of recommended engineering controls. These findings underscore the importance of institutional commitment and infrastructure in ensuring that PPE use is consistent and effective.

Although few data are available regarding potential contamination of family members of healthcare professionals handling cytotoxic drugs, the possibility of “take-home exposure” cannot be entirely excluded, further highlight the relevance of the use of PPE. Several studies have demonstrated exposure of relatives of oncology patients to antineoplastic agents [76,77,78]. While absorption and transfer of these agents from healthcare professionals to their households at concentrations comparable to those of patients is unlikely—since contamination in occupational settings typically occurs at residual levels far below therapeutic doses—the reality is that professionals may be exposed repeatedly and cumulatively over many years. This raises the need for greater awareness and further research into the possible indirect risks posed to the acquaintances and household members of oncology healthcare professionals.

### 4.2. Ergonomics

Ergonomics plays a critical but often underrecognized role in hazardous drug handling. Tasks such as drug compounding, patient care, and cleaning require repetitive movements, static postures, and high cognitive demand—conditions conducive to musculoskeletal disorders (MSDs). Multiple studies reported high prevalence rates of MSDs among healthcare workers, especially in the lower back, shoulders, neck, and upper limbs [64,66].

Manual compounding workstations are often not ergonomically designed. As noted by McLeod et al. [56], manual compounding sessions had significantly higher upper limb disorder scores than automated systems. Similarly, Villain et al. [74] found that automation significantly reduced MSD risks, especially in the shoulders, wrists, and fingers.

Organizational ergonomics is equally essential. Poor workflow design, staff shortages, and lack of standard procedures were repeatedly cited as contributors to physical and cognitive overload [6,69]. For instance, nurses in under-resourced facilities reported emotional and physical fatigue, often exacerbated by high patient volumes and poor team support [62,65].

Even infrastructure elements affect ergonomic safety. For example, only 20% of facilities reported in the study by Kopp et al. [63] had designated cytotoxic drug preparation areas, leading to tasks being performed on therapy counters or multipurpose benches. These layouts increase both contamination and injury risk due to physical strain.

Preliminary evidence suggests that automation may help reduce ergonomic risks, although broader studies are needed to confirm its effectiveness across diverse settings. Villain et al. [74] and McLeod et al. [56] both demonstrated improved ergonomic conditions through automation, including reductions in static posture duration and repetitive movements. However, these systems are costly and require standardization, which limits widespread adoption, especially in lower-resourced settings.

### 4.3. Training Gaps

The consistent theme across studies is a disconnection between knowledge of PPE/ergonomics and actual behavior. Nurses, pharmacy technicians and pharmacists often demonstrated high theoretical awareness but failed to translate this into consistent safe practices [6,62]. In the studies included in this review, the evaluation of the impact of PPE use and/or ergonomics in handling cytotoxic drugs was most frequently reported in relation to pharmacy technicians (*n* = 8) [6,65,67,68,69,71,73,74] and nurses (*n* = 5) [6,33,62,65,73]. This finding highlights the central role of these professionals in cytotoxic drug handling and, consequently, their greater likelihood of experiencing both chemical and ergonomic occupational exposures. Indeed, for different reasons, these two professional groups are at heightened risk of developing adverse health effects or musculoskeletal disorders. Nurses, for instance, have direct contact with patients, which generates significant ergonomic challenges [79,80], and often handle cytotoxic drugs in clinical settings where primary engineering controls, such as laminar airflow hoods, are not available [81]. Pharmacy technicians, on the other hand, face ergonomic challenges related to repetitive and precise movements carried out for prolonged periods in restricted spaces [65]. From a chemical exposure perspective, although their work environments are generally better controlled than those of nurses (e.g., use of laminar airflow hoods), pharmacy technicians handle the concentrated formulations of cytotoxic drugs, whereas nurses usually work with diluted solutions [82]. For any of the mentioned professional groups, the aforementioned training gap may be attributed to a lack of time, institutional support, or practical training.

Hands-on training is especially important. Kopp et al. [63] reported that only 25% of facilities provided practical instruction, even though 83% had some form of training program. Similarly, Gonçalves et al. [69] observed that 80.7% of handlers lacked institutional training, which correlated with higher accident rates and improper PPE use.

This issue is not exclusive to PPE. Ergonomic training and assessments are virtually nonexistent across many healthcare facilities. Most ergonomic findings in the reviewed studies were inferred from infrastructure and workflow design rather than direct assessment or staff feedback [29,65].

This lack of institutional engagement compromises safety culture. As noted by McDiarmid & Condon [72], poor enforcement of safety guidelines and the absence of feedback loops reduce PPE adherence and increase exposure risks. Facilities with stronger safety climates consistently showed better compliance and fewer adverse health effects.

### 4.4. Psychosocial and Organizational Factors

The ergonomic risks extend beyond physical strain to psychosocial stressors, including long working hours, shift work, patient suffering, and mental exhaustion. These factors contribute to burnout and can impair judgment, increasing the risk of procedural errors [64,65].

In addition, many workers operate under cognitive overload due to multitasking, poor workflow design, and a lack of breaks [66,71]. High-risk tasks like cytotoxic drug preparation often demand concentration and precision, making cognitive fatigue particularly hazardous.

The organizational environment plays a pivotal role. A culture of safety, supported by regular training, transparent incident reporting, and ergonomic design, can significantly reduce exposure and injury rates [69,72]. Unfortunately, many settings lack such systemic support, especially in low- and middle-income countries where resource limitations exacerbate risks [66].

### 4.5. Engineering Controls and Recommendations

Engineering controls such as laminar flow hoods, biosafety cabinets, and CSTDs provide foundational protection and should be integrated with PPE use. Studies confirmed that when properly used, these systems reduce environmental and personal contamination [29,67]. Yet, inconsistencies in maintenance and usage compromise their effectiveness [71].

The adoption of CSTDs, though effective, remains uneven due to cost, lack of training, and resistance to workflow changes. Tang et al. [67] found that CSTDs improved both safety and ergonomic satisfaction, while Bhirich et al. [71] highlighted their role in minimizing aerosolized contamination during drug transfer.

Moreover, engineering controls can ease ergonomic strain. Adjustable workstations, mechanical aids for transport, and automation of repetitive tasks reduce the need for prolonged static postures and awkward movements [64,74].

To maximize safety, PPE must be viewed as part of a broader safety system—complemented by engineering controls, administrative policies, and a proactive safety culture [57,72]. Isolated reliance on PPE, especially when misused or poorly selected, offers insufficient protection against cytotoxic drug exposure.

### 4.6. Research Gaps, Practical Implications, and Future Directions

This review also highlights important gaps in the existing literature. Most studies have been conducted in high-income countries, while evidence from low- and middle-income countries remains scarce, despite these settings often facing greater structural and financial barriers to implementing safety measures. Furthermore, the majority of available studies rely on cross-sectional and observational designs, limiting the ability to establish causal links between protective practices, ergonomic conditions, and health outcomes. Longitudinal studies are particularly lacking, which prevents a comprehensive understanding of the long-term impact of occupational exposure and ergonomic risks on healthcare professionals.

From a practical perspective, the findings underscore the need for institutional commitment in three key areas. First, continuous and hands-on training should be prioritized to ensure correct use of PPE and adoption of ergonomic strategies. Second, healthcare organizations should implement clear policies regarding PPE replacement, environmental monitoring, and psychosocial support, reinforcing a culture of safety. Third, investment in ergonomic infrastructure and technological solutions—such as adjustable workstations, closed-system transfer devices, and automated compounding systems—should be encouraged, as these interventions can simultaneously reduce chemical exposure and musculoskeletal strain.

Looking ahead, future research should include well-designed intervention studies assessing the effectiveness of structured training programs, ergonomic redesign of workspaces, and adoption of automation technologies. Cost–benefit analyses of protective measures are also warranted, as they can demonstrate the broader economic advantages of investing in worker safety, including reductions in absenteeism, occupational illness, and staff turnover. By addressing these gaps, the field can move towards evidence-based strategies that improve both worker protection and organizational performance.

### 4.7. Limitations

This review has several limitations that should be acknowledged. First, the number of included studies was relatively small, and many of them relied on cross-sectional or self-reported data, which introduces risks of recall and reporting bias. Second, the heterogeneity of study designs, populations, and outcome measures limited the possibility of direct comparisons or meta-analytic synthesis. Third, our search was restricted to three major databases (PubMed^®^, Google Scholar^®^, and Web of Science^®^). Although these are widely used sources, it is possible that relevant studies indexed elsewhere were not captured, particularly those published in non-English journals or reported in the gray literature. Finally, the geographical distribution of the included studies was skewed towards high-income countries, reducing the generalizability of findings to low- and middle-income settings. These limitations suggest that our synthesis, while informative, should be interpreted with caution.

## 5. Conclusions

The safe handling of cytotoxic drugs hinges on the effective and consistent use of PPE in conjunction with sound ergonomic practices. While PPE remains a necessary line of defense, it is only effective when selected appropriately, used correctly, and supported by systemic measures including training, engineering controls, and organizational commitment. Ergonomic risks—particularly musculoskeletal and cognitive stress—remain under-addressed but are critical to long-term health outcomes. A multidimensional safety strategy is essential to protect healthcare workers from the complex hazards posed by cytotoxic drug handling.

## Figures and Tables

**Table 1:** Search strings used in each database.

Database	Search String
PubMed	(“Cytotoxic Drugs”[MeSH Terms] OR “Antineoplastic Agents”[MeSH Terms] OR “Hazardous Drugs”) AND (“Handling” OR “Exposure”) AND (“Ergonomics” OR “Musculoskeletal Injuries” OR “Personal Protective Equipment” OR PPE)
Web of Science	Cytotoxic Handling Musculoskeletal Injuries Personal Protective Equipment Ergonomics
Google Scholar	Cytotoxic Handling Musculoskeletal Injuries Personal Protective Equipment Ergonomics

**Table 2:** Characteristics of the observational and experimental studies included in the review, detailing study type, country, journal, year of publication, and sample characteristics.

Reference	Year of Publication	Study Type	Country	Journal	Sample	Limitations
Fransman et al. [73]	2005	Experimental	The Netherlands	*Int Arch Occup Environ Health*	Total of 389 samples of dermal exposure from healthcare professionals (pharmacy technicians, oncology nurses and cleaning personnel), surfaces and materials (e.g., gloves) from four hospitals	The entire glove was analyzed; contamination might have been on the inside, indicating actual rather than potential exposure. Recovery efficiency was assumed equal across different glove types (based on a prior study with only one glove brand), possibly causing exposure misclassification. Multiple dermal exposure assessment techniques were used (e.g., glove analysis, skin wipes), each with differing efficiency. Surrogate sampling methods (e.g., gloves and patches) may overestimate exposure, particularly on forearms. Hospital layout differences (e.g., cleaning the whole bathroom vs. just the toilet) influenced exposure results.
Wallemacq et al. [61]	2006	Experimental	The medical school of the Catholic University of Louvain in Brussels, Belgium.	*Am J Health Syst Pharm*	Different glove types: Neoprene, nitrile, and natural rubber latex gloves. Total of 13 gloves testing 13 cytotoxic agents used by pharmacy personnel (undisclosed professions)	Lab-based exposure conditions (controlled temperature and time) may underestimate real-life permeation, as higher temperatures (body heat), motion, and humidity increase risks. No standardized prediction possible: Permeation varied significantly even between gloves of the same material and thickness from different manufacturers. Inactive drug ingredients and glove manufacturing processes affect outcomes. Only permeability was tested—no data on worker exposure or contamination rates in clinical settings.
Kosgeroglu et al. [62]	2006	Cross-Sectional Observational	Hospitals in Eskisehir, west Turkey	*J Clin Nurs*	A total of 121 nurses responded to a questionnaire concerning exposure to chemotherapy	Study only included 121 nurses from Turkey, limiting its generalizability Observational bias may exist as behavior was partly self-reported
Yoshida et al. [33]	2008	Cross-sectional Observational	Osaka Prefecture, Japan	*J Occup Health*	Total of 107 questionnaires answered by doctors, nurses and pharmacists, depicting the conditions of preparation of cytotoxic drugs, in their respective hospitals	Self-reported data gathered through questionnaires may be subject to bias or underreporting. No statistical analysis was conducted to compare awareness levels by job role due to insufficient sample size. Exposure levels were not measured directly (no environmental sampling or biological monitoring). Waste and excreta handling practices were poorly documented and implemented, especially regarding patient excreta, which may pose delayed exposure risks.
Constantinidis et al. [6]	2011	Cross-sectional Observational	Greece	*Eur J Cancer Care (Engl)*	Questionnaire distributed by 24 Greek hospitals with 353 answers by healthcare workers (96% corresponding to assistant nurses, and the remaining to pharmacist assistants and technicians) enrolled in handling chemotherapeutic drugs	Most data were collected through self-assessment questionnaires, introducing possible subjectivity or recall bias. Reported symptoms (e.g., headaches, skin irritation) were not clinically diagnosed. Causal relationships between exposure, PPE use, and health outcomes cannot be firmly established Lack of institutional health and safety infrastructure (e.g., occupational physicians, safety officers) reduced formal evaluation or reporting mechanisms.
McLeod et al. [56]	2012	Prospective Observational	United Kingdom hospital pharmacy	*Eur J Hosp Pharm*	Observation of 21 manual preparation sessions of cytotoxic drugs by 6 operators and 4 automated preparation sessions by 2 operators (exact professions undisclosed)	Small sample size, particularly for automated sessions (*n* = 4), limits generalizability. Sessions were not matched for number/type of drugs compounded—ULD risk likely increases with volume and complexity. Observer bias risk due to long observation periods (up to 148 min); inter-rater reliability could not be statistically confirmed. Psychosocial risk factors were not included in the ART assessment, although these are known contributors to ULDs.
Kopp et al. [63]	2013	Cross-sectional Observational and Experimental	Germany	*Int Arch Occup Environ Health*	Forty institutions (17 day hospitals and 23 private clinics) completed a questionnaire on cytotoxic handling A total of 375 surface wipe samples were collected from 28 facilities, including floors of medical and therapy rooms, toilets, infusion poles, and infusion pumps, testing for 8 cytotoxic agents.	Only 40 out of 137 contacted facilities participated, potentially introducing selection bias. Small sample size for correlation analysis limited statistical power. Cross-contamination and cleaning variability: Difficult to attribute contamination to specific work practices.
Viegas et al. [29]	2014	Experimental	Portugal	*Environ Monit Assess*	A total of 327 surface wipe samples were collected from 2 Portuguese hospitals, testing for 3 cytotoxic agents, prepared by pharmacy technicians.	The study is based on only two hospital settings, which limits the generalizability of the findings. Lack of detailed reporting on PPE practices: while environments imply proper use, the actual compliance, comfort, or training aspects are not explored. Ergonomic assessment is indirect—findings are inferred from infrastructure and workflow design rather than direct ergonomic evaluation or staff feedback. No quantitative data on incidents, exposures, or ergonomic strain are provided, limiting statistical analysis.
Villain et al. [74]	2020	Experimental and Cross-sectional Observational	France	*Pharmaceutical Technology in Hospital Pharmacy*	Comparison of 24 measurements in manual filling and 24 in automated mode Six pharmacy technicians completed a questionnaire for each of the two elastomeric pumps, both with automated and manual filling (24 questionnaires in total)	Calibration method depends on visual reading by staff and is operator-dependent, requiring strict training to minimize risk—volume delivery accuracy may be affected by poor calibration, although mitigated by a gravimetric control system with a 5% detection threshold. Economic limitation: Automation introduces a higher upfront cost, though this cost decreases significantly when multiple preparations are made. Cost-effectiveness depends on routine use and may improve with bulk pricing negotiations.
Rocha et al. [65]	2021	Cross-sectional Observational	Brazil.	*Rev Bras Med Trab*	A total of 40 healthcare workers handling antineoplastic drugs (11 nurses, 14 pharmacists, and 15 technicians) from the 3 major referral centers for chemotherapy treatment	Small sample size (*n* = 40) from only three referral centers in a single city (Porto Alegre), limiting generalizability. Self-reported data on accidents and PPE use may introduce recall or reporting bias. Underreporting of accidents: Only 44.4% of those who experienced exposure submitted official Work Accident Statements. No direct ergonomic assessments (e.g., posture analysis, workload evaluation) were performed—risks were inferred rather than measured. Lack of documentation of training activities limits the ability to assess the effectiveness of safety programs. Knowledge gaps among healthcare workers suggest training content may be insufficient or inconsistently delivered.
Tang et al. [67]	2022	Experimental	China	*Front Public Health*	A total of 96 wipe samples were collected from protective equipment (gloves, masks) and other sites (door handles, air-intake vents, transfer containers, etc.) in 1 hospital in Shanghai, testing for 2 cytotoxic agents, handled by 8 pharmacists and 6 pharmacy technicians.	Single-center study, which limits the generalizability of the findings. Small sample size, potentially reducing the statistical power and robustness of the results.
Gonçalves et al. [68]	2022	Cross-sectional Observational	Portugal	*Procedia Computer Science*	A questionnaire was applied to 83 professionals involved in cytotoxic handling (“most pharmacy technicians”—undisclosed percentage or representation by other professions) from 18 hospital institutions	Exploratory and descriptive design; lacks analytical or inferential scope Included studies collected data restricted to specific hospitals, limiting international generalizability, and displayed high difficulty obtaining responses, leading to potential non-response bias.
Gonçalves et al. [69]	2023	Cross-sectional Observational	Portugal	*Procedia Computer Science*	A questionnaire was applied to 83 professionals involved in cytotoxic handling (“most pharmacy technicians”—undisclosed percentage or representation by other professions) from 18 hospital institutions	Low response rate from hospitals hindered data collection and required persistent follow-up from researchers—Limited generalizability of the findings to the entire population of cytotoxic handlers. Potential selection bias, as not all invited professionals participated. Use of self-reported questionnaires, which may be subject to underreporting or recall bias
Zhou et al. [70]	2023	Experimental	China	*J Oncol Pharm Pract*	A total of 240 samples from 5 different glove materials were tested for permeation of 8 cytotoxic drugs (no specific professionals involved as the study targeted the gloves)	High variability in repeated tests for the same glove/drug/time combination, possibly due to inconsistencies in glove material composition. The experiment was in vitro; actual clinical conditions (e.g., hand movement, temperature, sweat, and friction) were not simulated. Only single-use gloves were tested per trial, limiting real-world generalizability where gloves may be worn for extended periods. The solvent used (ethanol) may have enhanced drug permeation in certain cases (e.g., DCT, ETP), limiting applicability to drugs prepared with different solvents. Ergonomic and psychosocial factors related to glove use (e.g., discomfort, fatigue) were not addressed. The study was conducted in a controlled laboratory setting, not in a live clinical environment, which may limit extrapolation of results.
Bhirich et al. [71]	2023	Qualitative Risk Analysis/Observational Study	Morocco	*J Oncol Pharm Pract*	Multidisciplinary group of 7 pharmacy professionals (1 professor of pharmacotechnics, 3 resident pharmacists, 1 pharmacist in charge of unit of cytotoxic preparation, and 2 pharmacy operators)	The FMECA analysis was based on subjective assessments from a multidisciplinary working group; ratings of severity, frequency, and detectability may vary with different participants. No quantitative exposure measurements (e.g., surface or biological contamination) were performed; results are based on perceived risk, not direct monitoring. The study was single-centered, conducted in a single institution, limiting generalizability. Despite identifying 12 failure modes, only three were deemed critical, and their CI was partially influenced by group discussion biases.

ART—Assessment of Repetitive Tasks; CI—Criticality Index; DCT—Docetaxel; ETP—Etoposide; FMECA—Failure Modes, Effects, and Criticality Analysis; PPE—Personal protective equipment; ULD—Upper limb disorder.

**Table 3:** Characteristics of the review studies included in the review, including review type, journal, number of studies included, languages of eligible studies, year of publication, and databases searched.

Reference	Year of Publication	Review Type	Journal	Number of Included Studies	Languages of Included Studies	Searched Databases	Limitations
McDiarmid & Condon [72]	2005	Narrative Review	*Journal of Occupational and Environmental Medicine*	Not revealed	Not revealed	Not revealed	There is a lack of systematic implementation of safety standards and feedback mechanisms.
Che Huei et al. [64]	2020	Systematic Review	*SAGE Open Medicine*	30	Not revealed	MEDLINE (Ovid), PubMed, PMC, TOXLINE, CINAHL, PLOS One, and Access Pharmacy	Selection bias: although published studies and some of the gray literature were included, potentially valuable unverifiable reports may have been excluded. Reliance on observational studies: no intervention studies were included, which may limit causal inferences. Heterogeneity of included studies: variability in methodologies across the 30 studies raises challenges for standardization and comparison.
Rai et al. [66]	2021	Scoping Review	*Int J Environ Res Public Health*	99	English	Five electronic databases using MEDLINE, Scopus, CINAHL, Embase, and PsycINFO	Very few studies focused specifically on LMIC healthcare workers, despite the high burden of MSDs observed. Use of different sampling methods (gloves, handwash, wipes, patches) with variable efficiencies; surrogate methods may overestimate exposure. Possible overestimation of glove protection due to sampling entire glove (both inside and outside contamination included).
Moreira et al. [57]	2025	Systematic Review	*J Am Pharm Assoc*	20	English, Spanish, or Portuguese	PubMed, Cochrane, and LILACS (Literatura Latino-Americana e do Caribe em Ciências da Saúde)	Heterogeneity in the scope, structure, and content of the reviewed guidelines limited comparability. Most guidelines lacked detailed educational content or operational definitions regarding training. Limited geographic representation, with an overrepresentation of high-income countries. The assessment relied heavily on AGREE II scores, which may not capture all nuances of guideline applicability.

LMIC—low-income or middle-income countries; MSD—Musculoskeletal disorders.

**Table 4:** Main findings from the studies included in this review regarding the use of personal protective equipment (PPE).

Reference	Key Findings on the Use of PPE
McDiarmid & Condon [72]	Historically, compliance with PPE use has been inconsistent and suboptimal. Gloves were the most used PPE, but even their use was low in early decades. Use of masks, gowns, and goggles was significantly lower than recommended. Although glove usage reached near 100% in recent years, usage of other PPE still falls below OSHA guidelines. Lack of availability of PPE; poor training and education regarding hazardous drugs risks and correct PPE practices; and inadequate written protocols are important barriers to PPE use
Fransman et al. [73]	Pharmacy technicians always wore gloves (mostly latex), with 27% using double gloving. Gloves generally provide good protection, with minimal contamination of hands underneath. Oncology nurses had inconsistent glove use: 100% wore gloves when handling patients’ urine, yet hands were still contaminated—suggesting poor glove integrity or accidental exposure. Only 36% used gloves while washing patients and 29% when removing bed sheets, resulting in higher dermal exposure. Cleaning personnel wore gloves (26% nitrile, 74% latex; 26% used double gloves). Gloves effectively prevented hand contamination despite high surface contamination on cleaning materials. Regarding other PPE: Pharmacy technicians also used aprons, surgical masks, and hair covers. Head and forearm contamination was occasional but present, likely due to lack of full-body coverage.
Wallemacq et al. [61]	Neoprene, nitrile, and certain natural rubber latex gloves provided the highest resistance to cytotoxic drug permeation. Vinyl gloves were the most permeable, with some showing unacceptable permeability within 15 min for multiple drugs. Permeation rates increased over time, with a mean 5-fold increase from 15 to 60 min. Some drugs (e.g., cyclophosphamide, docetaxel) showed a 10-fold increase. Thicker gloves (≥0.24 mm for natural rubber, ≥0.16 mm for nitrile) showed better protection, whereas the thinnest glove (vinyl, 0.12 mm) had the highest permeability. Carmustine was the most permeative drug, affecting various glove materials. ASTM D6978-05 permeation limit (10 ng/cm^2^/min) was exceeded by several gloves for several drugs, especially with vinyl and thinner gloves. Gloves should be changed at least every 30 min, ideally every 15–20 min when handling cytotoxic agents.
Kosgeroglu et al. [62]	Although nurses demonstrated high awareness of safety protocols, actual use of PPE was consistently lower. Although 74.8% of the participants knew gloves should be discarded in special containers, only 57.6% did so in practice. Other PPE components (e.g., masks, goggles, aprons) showed low use rates.
Yoshida et al. [33]	Availability and usage rates of PPE was very variable with gloves being the most frequently used item—Gloves (82.7%), masks (69.0%), gowns (62.1%), goggles (36.8%). Double gloving was practiced in 29.2% of hospitals. 10.1% of hospitals reported no PPE usage at all. Goggles underused despite the risk of ocular contamination. Discomfort while wearing goggles was cited as a common reason for non-use. Spill kits and sterilized sheets were more frequently used in hospitals where staff wore double gloves. Small-scale hospitals showed significantly lower availability and adoption of PPE
Constantinidis et al. [6]	While most employees reported using PPE, many used only gloves. Masks, gowns, goggles, caps, and shoe covers were used sporadically. PPE use was higher among those involved in preparation/reconstitution than in transportation/storage, likely reflecting better awareness or training. Only 51% of those handling drug preparation used gloves specifically designed for cytotoxic agents, despite evidence that glove material and thickness influence permeability. Morning-shift workers were more likely to comply with PPE use than shift workers. Many workers did not receive formal training; PPE use often correlated with experience or workload rather than age or education. No clear correlation with health outcomes: Although adverse health effects were reported, they did not statistically correlate with PPE use—possibly due to the inadequacy of the PPE used (only partial protection).
Kopp et al. [63]	Glove use was high but inconsistent: 92.5% of staff wore gloves during drug administration; however, only 17.5% wore gloves when unpacking transport boxes, and 17.5% did not wear them at all during this task. Glove use was sporadic during critical tasks such as connecting infusion sets (17.5%) and cleaning (2.5%). Limited use of additional PPE: Protective garments (e.g., scrubs or coats) were used in 65.5% of cases. Impermeable gowns, goggles, and masks were used only in response to spills or during drug preparation.
Viegas et al. [29]	Although PPE usage is not described in detail, the infrastructure implies mandatory use of gloves, gowns, masks, and other barrier protections in the cleanroom environments The highest contamination of paclitaxel was observed during drug transportation, likely due to gloves and metal trays.
Che Huei et al. [64]	A range of PPE types are discussed across hazards: face shields, goggles, surgical masks, respirators, gowns, gloves, and protective clothing. PPE is consistently recommended for chemical, and physical hazards, including aerosolized drug handling and needle-stick incidents. PPE effectiveness is emphasized but considered the least effective control in the hierarchy, especially when used in isolation. It is recommended in conjunction with engineering and administrative controls. PPE usage is tied to proper risk assessments and training, highlighting the importance of education on selection and correct usage.
Rocha et al. [65]	High exposure rates to cytotoxic drugs were reported: 67.5% of participants had experienced some form of occupational accident related to antineoplastic drugs. Contact exposure was the most common route (96.3%)—further highlighting the relevance of the use of PPE—followed by aerosol (14.8%), ingestion (3.7%), and inhalation (3.7%). A significant proportion of workers were not using PPE correctly during observed work activities.
Rai et al. [66]	Included studies with pharmacy technicians from LMIC revealed the frequent use of gloves, aprons, surgical masks, and hair covers during cytotoxics’ preparation. Although gloves were highly contaminated, skin underneath was rarely contaminated, indicating good protection. As to PPE use among nurses, it varied greatly by task (handling urine; patient washing; remotion of bed sheets). Gloves sometimes failed to protect hands; hand contamination occurred even without glove surface contamination, raising concerns about glove integrity or improper donning/doffing. Regarding cleaning personnel, it was described as a constant use of gloves, although the use of double gloves was uncommon.
Tang et al. [67]	CSTD significantly reduced contamination with cyclophosphamide and cytarabine on surfaces and PPE compared to traditional systems.
Gonçalves et al. [68]	High perceived importance of some PPE items, according to some studies: disposable caps; P3 masks, or, alternatively P2 masks; sterile latex gloves or, at a less extent, other glove types (nitrile, thick latex) Certain PPE undervalued: goggles and surgical masks deemed not relevant or minimally important by many Non-optimal practices identified: Many professionals still use surgical (P1) masks, contrary to guidelines. Some PPE reused (mask, gown, cap), mainly due to perceived stability and cost-saving. Most studies reported the use of two pairs of gloves, changed hourly or after contamination events. PPE comfort is a key criteria for the selection of PPE and involvement of pharmacy services in PPE choice
Gonçalves et al. [69]	Handlers using P2 class masks reported fewer health symptoms. There is a statistically significant association between the type of mask used and the presence of health effects. A lack of institutional training (80.7%) may contribute to improper or non-use of PPE, increasing the risk of intoxication, infertility, and other adverse effects.
Zhou et al. [70]	Results demonstrated significant differences in protective capacity among glove materials: MTX, EPI, and VCR did not permeate any glove type, even after 180 min (Grade A protection). DCT and ETP showed the highest permeation rates, especially through RB gloves. CPE and PVC gloves showed the most consistent and effective protection across multiple drugs. Double-layer glove combinations (e.g., CPE + PVC, CPE + NT) improved protection and are recommended for clinical use, though attention is needed for 5-FU. The study provides graded recommendations (A–F) for glove selection based on drug permeability, supporting evidence-based PPE decisions in hazardous drug handling.
Bhirich et al. [71]	The study highlighted multiple failure modes related to insufficient or incorrect use of PPE, such as: Contamination of personnel due to non-compliance with dressing protocols. Glove permeability to cytotoxic agents when inappropriate materials are used. Risk mitigation strategies proposed include: Use of CSTDs to minimize aerosol formation. Validated isolators and HEPA-filtered laminar flow hoods. Improved PPE training and adherence to dressing rules. Annual medical surveillance and clinical monitoring of exposed personnel.
Moreira et al. [57]	Some guidelines explicitly recommend the use of PPE such as gloves, gowns, and respiratory protection during the preparation and administration of cytotoxic drugs. The use of CSTD devices is also emphasized in certain recommendations as a complement to PPE. Regular training on proper donning, doffing, and disposal of PPE is cited as essential to protect healthcare workers.

CPE—Chlorinated Polyethylene; CSTD—Closed system transfer devices; DCT—Docetaxel; EPI—Epirubicin; ETP—Etoposide; HEPA—High Efficiency Particulate Air; LMIC—low-income or middle-income countries; MTX—Methotrexate; NT—Nitrile; OSHA—Occupational Safety and Health Administration; PPE—Personal protective equipment; PVC—Polyvinyl Chloride; VCR—Vincristine.

**Table 5:** Main findings from the studies included in this review regarding ergonomics.

Reference	Key Findings on Ergonomics
Wallemacq et al. [61]	Glove performance depends on real-world conditions: Factors such as temperature (30 °C on skin), friction, sweat, and rubbing increase permeation. Manual tasks, such as reconstitution and prolonged exposure, increase the risk of degradation and breakthrough. Workers handling hazardous drugs must balance protection with comfort and dexterity. Discomfort with goggles or thick gloves may reduce compliance.
Kosgeroglu et al. [62]	Nurses worked 43.1 ± 3.6 h/week and cared for ~24 patients each. High workload and understaffing may impair compliance. Working hours negatively correlated with protective behaviors: Self-protection scores dropped as weekly hours increased (r = –0.535, *p* < 0.01). Resource gaps and lack of supportive infrastructure (e.g., availability of PPE, time for safe practices) were implied contributors to poor adherence.
Yoshida et al. [33]	Only 44.8% of hospitals used exclusive workbenches for antineoplastic drug handling. In 48.3% of hospitals, non-antineoplastic drugs were also handled on the same workbench. In 6.9%, even office work was performed on the same bench, increasing contamination risks. Biological safety cabinets were installed in only 57.4% of hospitals, and significantly fewer in small-scale hospitals. Unsafe ampoule handling led to injuries and possible drug vapor exposure.
Constantinidis et al. [6]	63.6% of employees cited poorly designed workspaces as a contributing factor to accidents. Accidents were more frequent in preparation/administration roles, likely due to high physical and cognitive demands. Workers identified poor ergonomics, time pressure, staff shortages, and high workload as major causes of incidents. Lack of laminar flow hoods, specialized preparation rooms, and appropriate transportation/storage equipment created additional ergonomic and safety risks.
McLeod et al. [56]	Manual compounding was associated with significantly higher upper limb disorder exposure scores (median 9.8) than automated compounding (median 2.5). Highest-risk domains in manual sessions included: Force exerted, arm movement, arm posture. Automated compounding eliminated all high-risk scores; all sessions were classified as low risk. Manual sessions lacked breaks, whereas automation allowed passive breaks during compounding cycles. Ergonomic benefits of automation include reduced force and repetition, improved posture, decreased physical strain, even in high-volume sessions Limitations in ergonomics still exist with automation: requires setup/cleaning, though no moderate/high ULD risks were associated with these tasks; restriction to infusion bags ≤500 mL and specific device types, necessitating manual compounding for more complex preparations
Kopp et al. [63]	Only 20% of facilities had specific zones for handling cytotoxic drugs, increasing the risk of exposure in multi-use areas due to the lack of designated work areas. In several settings, cytotoxic drugs were transported by hand rather than using trays, increasing physical strain and contamination risk. No mention of ergonomic training or assessment, suggesting this area may be largely overlooked.
Viegas et al. [29]	The clear division of responsibilities—pharmacy technicians for preparation and nurses for administration—helps optimize work roles and prevent cognitive overload or procedural errors, aligning with organizational ergonomics. Use of BSCs within Grade B rooms improves physical ergonomic conditions by centralizing high-risk activity to a controlled space.
Villain et al. [74]	Significant reduction in MSD risk with automated preparation compared to manual methods. Mean MSD risk score: - Manual filling: 23.5 (SD = 2.8) - Automated filling: 8.7 (SD = 4.5) Improvements observed in almost all evaluated body regions (shoulders, wrists, fingers, forearms), except for neck posture. Automation reduced repetitive motions, manual effort, and postural constraints.
Che Huei et al. [64]	Healthcare professionals commonly suffer musculoskeletal disorders, such as low back pain, wrist strain, and neck/shoulder pain. Risk factors include repetitive tasks. Poorly designed workstations, non-ergonomic equipment, and inadequate administrative support also constitute major risk factors. Engineering controls recommended: adjustable workstations and seating, mechanical aids, and automation where feasible. Long working hours, shift work, workplace violence, stress, and burnout were linked to fatigue, mental disorders, hypertension, and chronic illness and, thus, constitute psychosocial hazards that threaten organizational and cognitive ergonomics. Controls emphasized include improved scheduling, conflict training, and supportive organizational policies.
Rocha et al. [65]	The study identified the presence of ergonomic risks, defined as physical or mental strain caused by poor working conditions, which may lead to discomfort, illness, or musculoskeletal and psychological disorders. Nurses were highlighted as the group most exposed to physical and mental strain, due to both the nature of their tasks and emotional burden from patient care. Psychosocial risks (e.g., stress from patient suffering, shift work, and emotional fatigue) were recognized, which align with cognitive and organizational ergonomic concerns. There was no mention of ergonomic training, equipment design, or workflow adaptation, suggesting that ergonomic risks remain under-addressed in practice.
Rai et al. [66]	It was reported a high prevalence of MSDs: 12 out of 13 studies reported MSDs in at least one body region in the previous 12 months, with prevalence ranging from 50.7% to 95%. The most commonly affected site was the lower back (reported by 35.3–78.2% of participants). Other affected areas included: Neck: 28–49.8%; Shoulders: 23.5–52.1%; Upper back: 20.7–54%; Knees: 11–68.7% Identified occupational physical risk factors included: prolonged static postures, working in bent or twisted positions, lifting or transferring patients, managing high patient volumes, performing repetitive tasks Identified occupational psychosocial risk factors included: high stress and anxiety, mental exhaustion, poor workplace support, low decision-making autonomy, increased workload and monotonous tasks, lack of experience Geographical limitation: Very few studies focused specifically on LMIC healthcare workers, despite the high burden of MSDs observed.
Tang et al. [67]	Pharmacists using CSTDs reported higher satisfaction with ergonomics, encumbrance, and safety during the compounding process.
Gonçalves et al. [68]	Common self-reported organizational ergonomics and work conditions include: working in double-sided permeable work areas, laminar flow cabinets mostly vertical class II type B; disinfection performed before and after work; HEPA filters replaced mainly every 6–12 months by qualified technicians; aseptic rooms featuring air extraction/filtration and pressure control.
Gonçalves et al. [69]	Handlers working 7 h per day reported a higher perceived risk (median risk = 3.6 on a 1–4 scale). A high incidence of occupational accidents (80.7%) indicates potential failures in workflow design, physical layout, or ergonomic practices. Lack of continuous training and absence of standard operating procedures may compromise the proper adaptation of tasks to workers, a core principle of ergonomics. Perceived risk levels were high (mean score = 3.27 out of 4), especially for outcomes like mutagenicity, infertility, and cancer—reflecting mental workload and perceived insecurity (cognitive ergonomics).
Zhou et al. [70]	However, glove selection may influence manual dexterity, comfort, and tactile sensitivity, which are critical ergonomic considerations in clinical practice. The results may indirectly inform ergonomic practices by highlighting which glove types can provide both protection and likely maintain dexterity (e.g., avoiding bulky or permeable gloves that compromise task performance or comfort).
Bhirich et al. [71]	While ergonomics was not directly evaluated, several human factors and workflow elements emerged: Manual preparation under laminar flow hoods can expose staff to repetitive tasks and awkward postures. The need to maintain static positions for prolonged periods, particularly during complex reconstitution steps, can contribute to musculoskeletal strain. The team indirectly addressed ergonomic risks by recommending automation to reduce manual workload and improve safety. Stress and workload-related fatigue, may indirectly affect ergonomic health and performance.
Moreira et al. [57]	Ergonomics is not a primary focus of the guidelines analyzed; however, the importance of structured workflow and task allocation is mentioned in the context of reducing procedural errors and operator fatigue.

BSC—Biological Safety Cabinets; HEPA—High Efficiency Particulate Air; LMIC—low-income or middle-income countries; MSD—Musculoskeletal disorders; PPE—Personal protective equipment; ULD—Upper limb disorder.

## Data Availability

The original contributions presented in this study are included in the article. Further inquiries can be directed to the corresponding authors.

## Abbreviations

The following abbreviations are used in this manuscript:

ART	Assessment of Repetitive Tasks
BSC	Biological Safety Cabinets
COSHH	Control of Substances Hazardous to Health Regulations
CPE	Chlorinated Polyethylene
CSTD	Closed system transfer devices
DCT	Docetaxel
EMA	European Medicines Agency
EPI	Epirubicin
ETP	Etoposide
FMECA	Failure Modes, Effects, and Criticality Analysis
GMP	Good Manufacturing Practices
HEPA	High Efficiency Particulate Air
HSE	Health and Safety Executive
IARC	International Agency for Research on Cancer
ISOPP	International Society of Oncology Pharmacy Practitioners
LMIC	Low-income or middle-income countries
MTX	Methotrexate
MSD	Musculoskeletal disorders
NIOSH	National Institute for Occupational Safety and Health
NT	Nitrile
OSHA	Occupational Safety and Health Administration
PPE	Personal protective equipment
PVC	Polyvinyl Chloride
RB	Rubber-based
ULD	Upper limb disorder
VCR	Vincristine
